# Fusion Pore Diameter Regulation by Cations Modulating Local Membrane Anisotropy

**DOI:** 10.1100/2012/983138

**Published:** 2012-03-12

**Authors:** Doron Kabaso, Ana I. Calejo, Jernej Jorgačevski, Marko Kreft, Robert Zorec, Aleš Iglič

**Affiliations:** ^1^Laboratory of Biophysics, Faculty of Electrical Engineering, University of Ljubljana, 1000 Ljubljana, Slovenia; ^2^Laboratory of Neuroendocrinology-Molecular Cell Physiology, Faculty of Medicine, University of Ljubljana, 1000 Ljubljana, Slovenia; ^3^Departamento de Biologia e CESAM, Universidade de Aveiro, 3810-193 Aveiro, Portugal; ^4^Celica Biomedical Center, Tehnoloski Park 24, 1000 Ljubljana, Slovenia; ^5^Department of Biology, Biotechnical Faculty, University of Ljubljana, Vecna pot 111, 1000 Ljubljana, Slovenia

## Abstract

The fusion pore is an aqueous channel that is formed upon the fusion of the vesicle membrane with the plasma membrane. Once the pore is open, it may close again (transient fusion) or widen completely (full fusion) to permit vesicle cargo discharge. While repetitive transient fusion pore openings of the vesicle with the plasma membrane have been observed in the absence of stimulation, their frequency can be further increased using a cAMP-increasing agent that drives the opening of nonspecific cation channels. Our model hypothesis is that the openings and closings of the fusion pore are driven by changes in the local concentration of cations in the connected vesicle. The proposed mechanism of fusion pore dynamics is considered as follows: when the fusion pore is closed or is extremely narrow, the accumulation of cations in the vesicle (increased cation concentration) likely leads to lipid demixing at the fusion pore. This process may affect local membrane anisotropy, which reduces the spontaneous curvature and thus leads to the opening of the fusion pore. Based on the theory of membrane elasticity, we used a continuum model to explain the rhythmic opening and closing of the fusion pore.

## 1. Introduction

**Figure 1:**
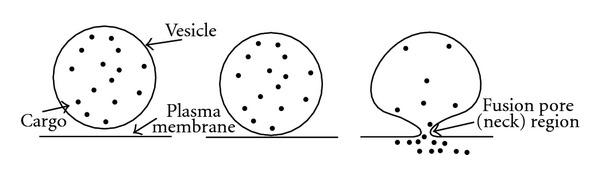
Schematic illustration of the vesicle fusion with the plasma membrane. Note that the fusion pore is a narrow membranous neck connecting the vesicle to the plasma membrane.

During exocytosis the membrane-attached vesicle can release its cargo through full fusion with the plasma membrane, or it can form a stable membranous neck (i.e., fusion pore) (Figure 1) mediating the cargo release via the repetitive fusion pore openings and closures [1, 2]. The advantages of the latter mechanism are that the vesicle can be reused in another round of exocytosis [3] and that the release process can be further modulated by the interaction between the vesicle lumen and the physical properties of the membranous neck region. The fusion pore is initially narrow (appears closed), and then it widens up to such an extent that the pore can be electrically measured as a transient increase in membrane capacitance. The time interval for the fusion pore opening or closure can range from milliseconds to minutes [4–6]. Recently, it has been shown that the rhythmic opening and closure of the fusion pore [4] can be further increased using a cAMP-increasing agent, which drives the opening of nonspecific cation channels. Yet, the underlying mechanisms for the enhanced fusion pore periodicity remain unclear. In the present paper, we constructed a computational model investigating the contribution of cation fluxes through the vesicle membrane and the fusion pore to explain the observed periodic transient fusion pore events induced by the application of a cAMP-increasing agent.

The energetics of the formation and stability of the fusion pore is not trivial. The increase of membrane bending energy, due to the transformation of the fusion pore into a thin tubular structure, is substantial [7, 8]. In addition, the narrow fusion pore increases the electrostatic repulsive forces between negatively charged lipids enriched in the opposing cell membranes [9]. On the other hand, SNARE (soluble N-ethylmaleimide-sensitive factor attachment protein receptor) proteins were suggested to facilitate the fusion of the vesicle with the plasma membrane [10, 11]. In addition, cholesterol and PIP_2_ lipids may form a nanodomain raft that stabilizes the highly curved fusion pore region [8, 12]. It was proposed that, without these stabilizing components, the fusion pore will spontaneously close or open irreversibly during complete exocytosis [13]. These stabilizing components may either increase the area difference between the two membrane leaflets or interact unequally with one of the membrane leaflets. Moreover, the increased stability could be due to the reduction in the curvature energy mismatch between the spontaneous curvature of the anisotropic membrane components and the tubular membrane curvature of the pore [8, 14–17]. The deviatoric theory of membrane elasticity has been previously employed to describe the stability of highly curved cell membrane geometries such as dimeric detergent-induced membrane nanotubes [18, 19], nanotubes of giant unilamellar vesicles [18, 19], cell membrane pores [20], narrow necks connecting daughter vesicles with the parent membrane [8, 21, 22], and inverted hexagonal phospholipid phases [23, 24]. The large difference between the two principal membrane curvatures of these structures increases the importance of anisotropic membrane components (either lipids, proteins, or their complexes) that have different spontaneous curvature along the two principal directions of the membrane [8, 22]. For example, a recent computational model has demonstrated that the narrow fusion pore can be reproduced only by the inclusion of anisotropic membrane components into the highly curved membrane nanodomain [8].

**Figure 2:**
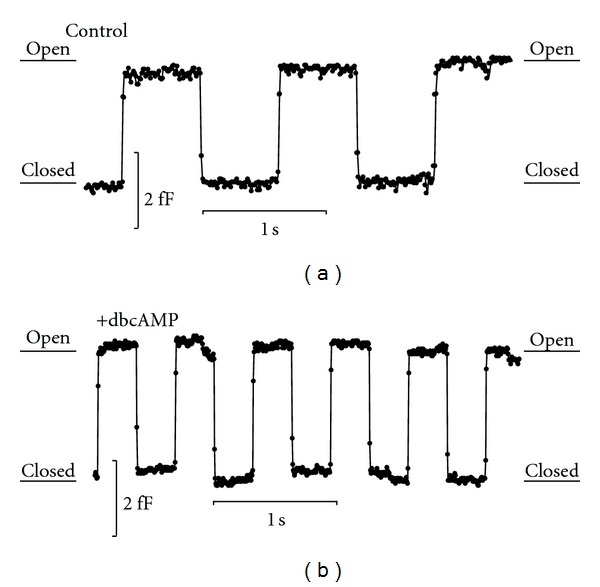
The application of a cAMP increasing agent increases the rhythmic openings and closures of an individual fusion pore, recorded in resting lactotrophs. Membrane capacitance recordings demonstrate the effects of a cAMP increasing agent (dbcAMP, 10 mM) (b) in comparison to the control experiment (a). The capacitance trace demonstrates changes between low and high states, corresponding to effectively closed and open fusion pore states.

**Figure 3:**
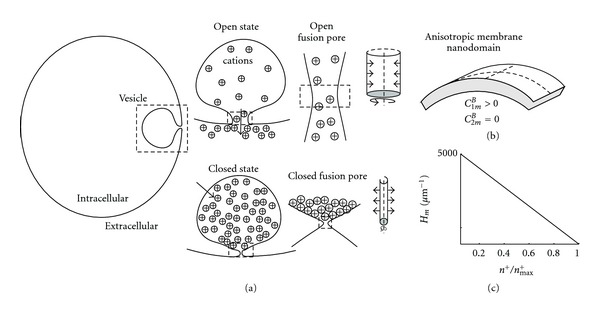
Schematic model for the rhythmic opening and closure of the fusion pore. The stable fusion pore of a vesicle can either be in open or closed states (a). The influx of cations via ion channels in the vesicle membrane is constant, while the efflux through the fusion pore out of the vesicle is allowed only when the fusion pore is open (see insets). The spontaneous curvature at the fusion pore is modulated by the cation concentration in the vesicle. The membrane force, derived from the differentiation of the system free energy, is exerted in the radial direction (see arrows) of the modelled axisymmetric fusion pore (see insets). The fusion pore is composed of a single anisotropic membrane domain (b). The membrane spontaneous curvature (*H*_*m*_) of the fusion pore is plotted as a function of the relative cation concentration (*n* ^+^/*n*_max⁡_^+^) (c). The proposed dynamics is that the cation flux through the vesicle increases the cation concentration in the vesicle, which then reduces the intrinsic membrane curvature (*H*_*m*_) leading to the opening of the fusion pore. The opening of the pore causes the abrupt reduction in cation concentration, which increases the spontaneous membrane curvature of the pore leading to the abrupt closing of the fusion pore.

The aim of the present paper is to study the underlying mechanisms responsible for the enhanced periodicity of fusion pore events following the application of a cAMP-increasing agent (Figure 2). We constructed a computational model, in which the fusion pore is modelled as a single anisotropic membrane domain (nanodomain). The membrane force exerted to open and close the fusion pore was derived from the differentiation of the membrane bending energy. The cation concentration in the vesicle was dependent on the morphologic features of the vesicle as well as the membrane area fraction of cation fluxes. The cation flux into the vesicle through the area fraction of cation channels increased the cation concentration at the closed (extremely narrow) pore, while the cation flux out of the vesicle through the pore was possible only when the pore was sufficiently wide (open). The rhythmic opening and closure of the fusion pore were reproduced by coupling the spontaneous curvature of the anisotropic membrane nanodomain and the cation concentration at the fusion pore (Figure 3). The physiological consequences of this novel model are discussed.

## 2. The Model

The aim of the present computational model is to explain the rhythmic opening and closing of the fusion pore (Figure 2). A vesicle is attached to the plasma membrane through the narrow fusion pore (Figure 1). It was reported previously that the diameter of the fusion pore may be <0.5 nm [5, 8]. In the model, the attached vesicle is composed of two compartments (Figure 3). The first compartment is a sphere, modelling the vesicle without the fusion pore. The second compartment is an axisymmetric tubular structure, modelling the fusion pore (neck) region. The dimensions of the vesicle and the fusion pore regions are according to previously published data [8]. It is assumed that there is a constant influx of cations through the vesicle membrane and that the efflux of cations occurs only through the fusion pore opening. The two membrane leaflets of the fusion pore are modelled as a single membrane entity. The diameter of the fusion pore is the only morphologic parameter being varied along the simulation. Previously, it has been shown that isotropic membrane components alone are not sufficient for the stability of the narrow fusion pore [8]. On the other hand, anisotropic membrane components are crucial for its stability [8, 21, 22]. For the sake of simplicity, in this work the membrane of the fusion pore includes only a single homogeneous anisotropic membrane domain. To summarize, the underlying assumptions in our model are as follows. (1)  The spontaneous curvature of the anisotropic membrane domain (nanodomain) is affected by the cation concentration in the vesicle. The accumulation of cations at the entrance of the fusion pore leads to lipid demixing, which reduces the spontaneous curvature of the anisotropic domain thereby opening the fusion pore. (2) The flux through the pore reduces the cation concentration in the vesicle, enabling the reorganization of the anisotropic membrane domain in the fusion pore and the increase in its spontaneous curvature, which closes the fusion pore (Figure 3).

### 2.1. Derivation of the System's Free Energy

The membrane bending (free) energy is considered within continuum approach [25–27]:

(1) $$\begin{array}{c} -F=\frac{\kappa }{4}\int \left[{\left(2H-2H_{m}\right)}^{2}+{\left(2D-2D_{m}\right)}^{2}+\sigma \right]dA, \end{array}$$

where *κ* is the membrane bending modulus, *H* = (*C*_1_ + *C*_2_)/2 is the mean membrane curvature, *D* = |*C*_1_ − *C*_2_ | /2 is the curvature deviator of the membrane, *H*_*m*_ is the spontaneous membrane curvature and *D*_*m*_ is the intrinsic (spontaneous) membrane curvature deviator, *C*_1_ and *C*_2_ are the principal membrane curvatures, *σ* is the surface membrane tension, and *dA* is the infinitesimal membrane area element. It can be seen from (1) that the membrane bending properties can be expressed in a simple way as a function of *H*_*m*_ and *D*_*m*_. The difference between the membrane curvature and the membrane intrinsic (spontaneous) curvature determines the energy cost for bending the membrane away from its favourable curvature. The cation concentration in the vesicle (*n* ^+^) affects the intrinsic membrane curvatures, as follows:

(2) $$\begin{array}{cc} H_{m} & ={\bar{H}}_{m}\left(1-\frac{n^{+}}{n_{\max}^{+}}\right),\\ D_{m} & ={\bar{D}}_{m}\left(1-\frac{n^{+}}{n_{\max}^{+}}\right), \end{array}$$

where ${\bar{H}}_{m}$ and ${\bar{D}}_{m}$ are the intrinsic membrane curvature and membrane curvature deviator, respectively, and *n*_max⁡_^+^ is the maximum concentration of cations. Note that the spontaneous curvature of the anisotropic membrane nanodomain depends on the normalized cation concentration (0 ⩽ *n* ^+^/*n*_max⁡_^+^ ⩽ 1).

The anisotropic membrane nanodomain has cylindrical intrinsic curvatures, where *C*_2*m*_ = 0 (see Figure 3(b)), and, consequently, ${\bar{H}}_{m}=1/2C_{1m}={\bar{D}}_{m}$. The radius of the fusion pore is

(3) $$\begin{array}{c} r\left(z\right)=R_{\text{eq}}+h\left(z\right) \end{array},$$

where *h*(*z*) is a small deviation from the initial equilibrium radius *R*_eq_ of the tubular fusion pore. More details on the curvatures of the tubular fusion pore are outlined in the Supplementary Material available online at doi:10.1100/2012/983138.

### 2.2. Equilibrium Model

The equation of motion of the membrane height deflection (i.e., in the radial direction) along the cylindrical main axis is given by [28, 29] the following

(4) $$\begin{array}{c} \varphi \frac{\partial h\left(z\right)}{\partial t}=-\frac{\partial F}{\partial h\left(z\right)}, \end{array}$$

where *φ* is the friction coefficient describing the drag of the fluid surrounding the membrane. The forces (per unit area) derived from the differentiation of the free energy (1) are outlined in the Supplementary Material. The fusion pore and the vesicle share the same cation concentration. As a result, the effects of cations on the spontaneous curvature of the fusion pore is uniform throughout the pore (Figure 3).

By a small perturbation to the uniform initial state, we obtain

(5) $$\begin{array}{cc} r\left(z\right) & =R_{\text{eq}}+\delta h\left(z,t\right), \end{array}$$

where *δh*(*z*, *t*) is a small deviation from the uniform value.

The linearization of (4) yields

(6) $$\begin{array}{cc} \varphi \frac{\partial h\left(z\right)}{\partial t} & =\int \left(U+\delta L\left(h\right)+O\left(\delta^{2}\right)\right)dA, \end{array}$$

where the function *δL*(*h*) describes the small undulation in the membrane force, and *O*(*δ* ^2^) describes higher-order terms of the membrane undulation. The force acting on the membrane at equilibrium state is described by *U*.

The equilibrium radius *R*_eq_ is obtained by assuming that the undulation *U* equals zero. The following equation was solved for *R*:

(7) $$\begin{array}{c} U=\varphi \kappa \frac{\left(-2+2{C_{1m}}^{2}{\left(n^{+}-1\right)}^{2}R^{2}+R^{2}\sigma \right)}{4R^{3}}=0. \end{array}$$

The resulted equilibrium radius of the fusion pore is

(8) $$\begin{array}{cc} & R_{\text{eq}}=\frac{1}{\sqrt{{{C_{1m}}^{2}\left(n^{+}-1\right)}^{2}+\sigma /2}}. \end{array}$$

### 2.3. Dynamic Model

The fusion pore is allowed to evolve only along the radial direction. We here consider the second layer of model complexity, which is the cation dynamics. The cation concentration is increased due to the influx through the vesicle membrane, while the efflux through the fusion pore reduces the cation concentration. The dynamics of cations is

(9) $$\begin{array}{cc} K_{\text{in}}= & 4\pi r_{v}^{2}A_{v},\\ K_{\text{out}}= & \pi R_{d}^{2},\\ \frac{\delta n^{+}}{\delta t}= & \left(K_{\text{in}}-{\text{K}}_{\text{out}}\right), \end{array}$$

where *t* is time, *K*_out_ and *K*_in_ are the flux rates through the fusion pore and vesicle, respectively, *A*_*v*_ is the (dimensionless) area fraction of the cation fluxes, and *r*_*v*_ and *R*_*d*_ are the radii of the spherical vesicle and the fusion pore, respectively. Note that *r*_*v*_ and *A*_*v*_ are defined at the start of the simulation, while *R*_*d*_ and *n* ^+^ are the two variables in the system. For the sake of simplicity, it is assumed that the cation concentration in the vesicle is equal to the cation concentration in the fusion pore.

## 3. Results

### 3.1. Equilibrium Shape Analysis

**Figure 4:**
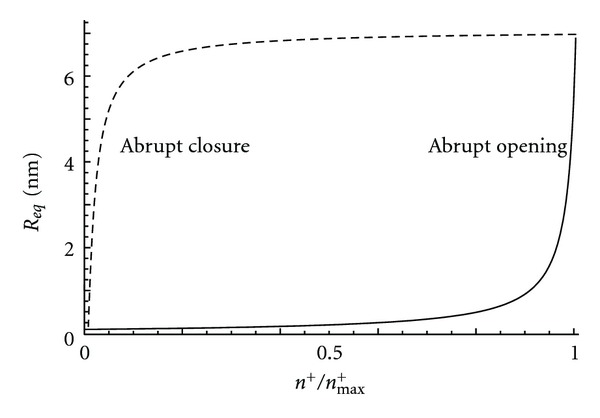
The relationship between the equilibrium radius of the pore (*R*_eq_) and the relative cation concentration in the vesicle (*n* ^+^/*n*_max⁡_^+^). The relationship between *n* ^+^/*n*_max⁡_^+^ and *R*_eq_ is derived from the minimization of the membrane bending energy. Note that an increase in the cation concentration (bold line) leads to an abrupt increase in the fusion pore radius, *R*_eq_ (i.e., the opening of the fusion pore), while, in the opposite direction (dotted line), a reduction in the cation concentration leads to an abrupt closure of the fusion pore.

We analyzed the effects of varying the relative cation concentration (*n* ^+^/*n*_max⁡_^+^) on the equilibrium radius (*R*_eq_). The parameter values used for the analysis were as follows: *C*_1*m*_ = 10000 *μ*m^−1^, *σ* = 0.001 g s^−2^, *r*_*v*_ = 100 nm, and *A*_*v*_ = 0.1. In Figure 4, we plot the relationship between *R*_eq_ and *n* ^+^/*n*_max⁡_^+^. In this relationship, the equilibrium radius was abruptly increased when *n* ^+^/*n*_max⁡_^+^ approached 0.9. The increase in the equilibrium radius was due to the reduction in the spontaneous curvature of the anisotropic membrane domain at the fusion pore. On the other hand, the reduction in the cation concentration from 0.9 to 0.1 led to the abrupt decrease in *R*_eq_ (i.e., the abrupt closure of the fusion pore).

### 3.2. Numerical Simulations: Dynamic Model

**Figure 5:**
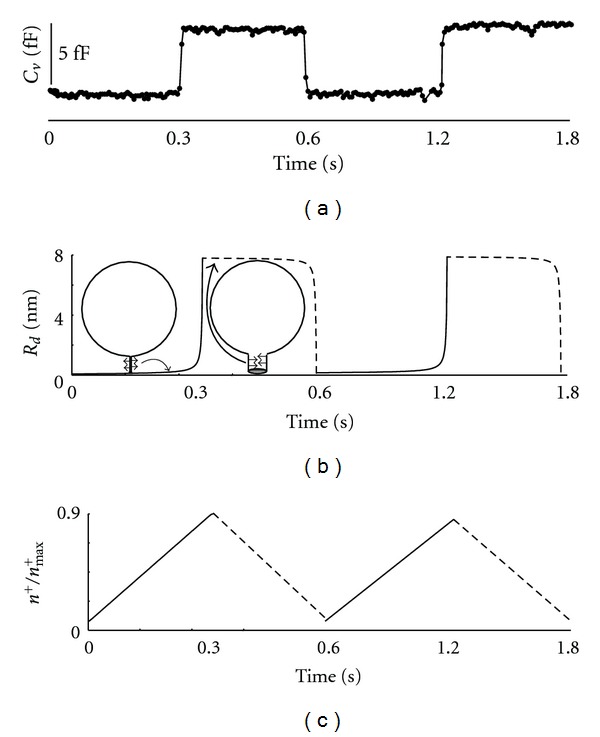
The effects of changes in cation concentration in the vesicle on the rhythmic opening and closure of the fusion pore. Cell-attached patch-clamp recordings (capacitance trace) following the application of a cAMP-increasing agent demonstrate fast rhythmic opening and closure of an individual fusion pore (a). Note that the capacitance change between closed and open fusion pore states is about 5 fF. Numerical simulations demonstrating the time dynamics of the pore radius (*R*_*d*_) (b). Note the abrupt opening and closure of the fusion pore. The membrane force derived from the membrane bending energy is exerted along the perpendicular direction to the major axis of the fusion pore (see arrows). The cation concentration dynamics includes the efflux through the fusion pore and the influx through the vesicle membrane (c).

In our dynamic model, the membrane force exerted along the perpendicular direction to the major axis of the fusion pore membrane was derived from the anisotropic membrane bending energy. To have a better understanding of how the cation fluxes through the vesicle membrane and fusion pore affected the opening and closure of the fusion pore, numerical simulations were employed to solve this nonlinear system of fusion pore dynamics. It was assumed that the forces exerted on the fusion pore membrane were the same in the open and closed states but in the opposite direction (Figure 4). In particular, the equilibrium pore radius diagram as a function of the cation concentration in the closed state of the pore was reversed to the one in the open state.  Figure 5 shows the functional dependence between changes in the fusion pore radius (Figure 5(b)) and cation concentration in the vesicle (Figure 5(c)). To obtain the same time interval for the open and closed states of the fusion pore, the cation flux rate through the fusion pore was set to be twice as large as the flux rate through the vesicle membrane. Note that the cation flux through the fusion pore effectively occurred only after its abrupt opening, while the cation flux through the vesicle membrane was active throughout the simulation. In the pore opened state, the cation concentration in the vesicle was increased from 0.1 to 0.9 over a time interval of 0.3 sec. In the pore closed state, the concentration of the cations in the vesicle was reduced from 0.9 to 0.1 over the same time interval.

## 4. Discussion

In this study, we have constructed a model of fusion pore dynamics to explain the enhanced periodicity in the opening and closure of individual fusion pores observed following the application of a cAMP-increasing agent (Figure 2). The assumed axisymmetric tubular fusion pore was connected to a vesicle of spherical geometry (Figure 3). The membrane of the fusion pore was composed of a single anisotropic membrane domain. The bending energy of the fusion pore membrane took into account the curvature mismatch between the spontaneous curvature of the anisotropic membrane pore domain and the curvature of the pore tubular membrane (Figure 3). The spontaneous curvature of the anisotropic membrane pore was considered to depend on the local concentration of cations. The differentiation of the free energy with respect to the membrane pore deformation gave a membrane force in the radial direction of the axisymmetric fusion pore. The influx of cations was through the membrane of the vesicle, while the cation efflux was through the fusion pore. In equilibrium state, the force exerted on the fusion pore membrane was zero, and the fusion pore diameter was mainly a function of the coupling between the intrinsic (spontaneous) curvature of the anisotropic membrane pore domain and the cation concentration (Figure 4).

Pituitary cells express various cation channels, for instance HCN channels which are modulated by cAMP [30]. These channels are permeable to Na^+^ and K^+^ and are therefore likely candidates for modulating local membrane anisotropy. Following the application of a cAMP-increasing agent, the patch-clamp membrane capacitance data from hormone secreting pituitary cells revealed faster rhythmic openings and closures of the fusion pore (Figure 2). To reproduce the sharp changes in the fusion pore diameter, it was necessary that the spontaneous curvature of the anisotropic membrane pore domain was sufficiently large. The influx of cations through the vesicle membrane (i.e., through ion channels) increased the cation concentration in the vesicle, which reduced the spontaneous curvature of the anisotropic membrane pore domain leading to the fast opening of the fusion pore. On the other hand, the cation efflux through the fusion pore reduced the cation concentration in the vesicle, which increased the spontaneous curvature of the anisotropic membrane pore domain causing the rapid closure of the fusion pore (Figure 5). To conclude, the complete cycle of fusion pore opening and closure could be driven by cation fluxes through the vesicle membrane and the fusion pore.

The fusion pore is a membranous neck of an attached vesicle with the plasma membrane (Figure 1). The stability of the fusion pore is evident from the periodic opening and closure of the same fusion pore [4–6]. The alternative scenario is the new formation of the fusion pore before each cycle of opening and closure, but this is of low probability due to the accompanied high bending energy costs [9]. By the cell-attached patch-clamp recordings in resting lactotrophs, the conductance of the fusion pore was estimated as low as a few pS, accounting for an equilibrium radius below 1 nm [5, 8]. In the present study, we revealed that the coupling of the pore membrane domain spontaneous curvature and the cation concentration may cause fast transitions between the open and closed fusion pore states.

To date, the composition of the proposed anisotropic pore membrane domain remains unclear. In general, the specific shape of fusion pore membrane can be stabilized by either lipids, proteins, or their complexes. Among the many possibilities, the SNARE protein complex is one of the major candidates responsible for the stability of the fusion pore. The SNARE protein complex is reported to direct the attachment of the vesicle to the plasma membrane, thereby overcoming the large energy barrier [9]. The SNARE protein complex at the plasma membrane is bound to synaptobrevin on the membrane of the vesicle [10]. This attachment has been shown to precede the formation of the fusion pore. The availability of synaptobrevin is regulated by sphingosine, a releasable backbone of sphingolipid [31]. Possibly, the electrostatic repulsion between sphingosines and positively charged residues of synaptobrevin could account for a change in synaptobrevin's structure enabling its contact with the SNARE protein complex [31]. However, the SNARE complex may change the fusion pore properties at the postfusion stage [8]. We here propose that the accumulation of cations at the entrance of the fusion pore (Figure 3(a)) may interact electrostatically with the positively charged sphingosines leading to the local dispersion (or demixing) of sphingosines in the anisotropic pore domain. The large anisotropic spontaneous curvature of the fusion pore could be stabilized also by negatively charged lipids such as PIP_2_ (Phosphatidylinositol 4,5-bisphosphate) lipids [12]. The aggregation of cations in the vesicle could attract PIP_2_ lipids from the pore region, thereby changing the local anisotropicity of the fusion pore.

Another possible mechanism for the stability and reversibility of the fusion pore is the dynamic reorganization of a cholesterol-sphingomyelin raft at the fusion pore. The raft is stabilized by hydrogen bonds and hydrophobic interactions between cholesterol and sphingomyelin lipids [32]. The anisotropic spontaneous curvature of the lipid raft might be, according to the molecular interactions, between the raft constituents. The narrow (closed) fusion pore could be stabilized by the gel-like properties such as increased rigidity of the cholesterol-sphingomyelin nanodomain raft. The increase in cation concentration may modulate the molecular interactions within the raft, thereby reorganizing the raft into a more disordered (liquid) state. The rapid and reversible phase transition between gel-like and disordered states may account for the rhythmic closing and opening of the fusion pore, respectively.

The proposed theory of fusion pore opening and closure dynamics awaits confirmation in future experiments. The usage of ion channel blockers against cAMP-channels may also modulate the rhythmic opening and closure of the fusion pore. The relationship between the fusion pore opening time interval (i.e., dwell time) and the number of cation channels can be investigated by the partial blocking of the ion channels. The origin of the anisotropic mechanical properties of the pore membrane can be identified by mutagenic assays and overexpression of specific lipids or proteins. Finally, we hope that this computational model would shed light on the intricate biophysical mechanisms underlying the rhythmic opening and closure of the fusion pore.

## Supplementary Material

Based on the theory of membrane elasticity, we use a continuum model to explain the rhythmic opening and closing of the fusion pore. The following is the derivation of the membrane force derived from the anisotropy of the fusion pore membrane. The equilibrium analysis performed in our study is described in more detail.
