# Supplementary material for: Fusion Pore Diameter Regulation by Cations Modulating Local Membrane Anisotropy
Source: ScientificWorldJournal. 2012 Mar 12;2012:983138. doi: 10.1100/2012/983138 (PMC3317573; doi:10.1100/2012/983138)
Supplement: Supplementary file 1 — Based on the theory of membrane elasticity, we use a continuum model to explain the rhythmic opening and closing of the fusion pore. The following is the derivation of the membrane force derived from the anisotropy of the fusion pore membrane. The equilibrium analysis performed in our study is described in more detail. [file 983138.f1.pdf]

# Supplementary material: Fusion pore diameter regulation by cations modulating local membrane anisotropy

Doron Kabaso, Ana I. Calejo, Jernej Jorgačevski, Marko Kreft, Rorbert Zorec, Aleš Iglič

December 29, 2011

Based on the theory of membrane elasticity, we use a continuum model to explain the rhythmic opening and closing of the fusion pore. The following is the derivation of the membrane force derived from the anisotropy of the fusion pore membrane. The equilibrium analysis performed in our study is described in more detail.

## Derivation of the curvature-dependent force

The membrane free energy of the system is [1, 2]:

$$F = \frac{\kappa}{4} \int [\,(2H - 2H_m)^2 + (2D - 2D_m)^2 + \sigma]dA \ , \tag{1}$$

where $\kappa$ is the membrane bending modulus, $H = (C_1 + C_2)/2$ is the mean membrane curvature, $D = |C_1 - C_2|/2$ is the curvature deviator of the membrane, $H_m$ is the spontaneous membrane curvature and $D_m$ is the intrinsic

(spontaneous) membrane curvature deviator, $C_1$ and $C_2$ are the principal membrane curvatures, $\sigma$ is the surface membrane tension, and $dA$ is the infinitesimal membrane area element. It can be seen from Eq.(1) that the membrane bending properties can be expressed in a simple way as a function of $H_m$ and $D_m$. The difference between the membrane curvature and the membrane intrinsic (spontaneous) curvature determines the energy cost for bending the membrane away from its favourable curvature. The cation concentration in the vesicle ($n^+$) affects the intrinsic membrane curvatures, as follows :

$$\begin{aligned} H_m &= \bar{H}_m(1 - n^+/n^+_{max}) \\ D_m &= \bar{D}_m(1 - n^+/n^+_{max}) \ , \end{aligned} \tag{2}$$

where $\bar{H}_m$ and $\bar{D}_m$ are the intrinsic membrane curvature and membrane curvature deviator, respectively, and $n^+_{max}$ is the maximum concentration of cations. Note that the spontaneous curvature of the anisotropic membrane nanodomain depends on the normalized cation concentration ($0 \leqslant n^+/n^+_{max} \leqslant 1$).

The anisotropic membrane nanodomain has cylindrical intrinsic curvatures, where $C_{2m} = 0$, and consequently, $\bar{H}_m = \frac{1}{2}C_{1m} = \bar{D}_m$.

The radius of the fusion pore is :

$$r(z) = R + h(z) \tag{3}$$

where $h(z)$ is a small deviation from the initial equilibrium radius $R$ of the tubular fusion pore.

## Membrane curvatures of the fusion pore

The surface of revolution of the fusion pore that has a tubular structure is defined by the two principal curvatures,

$$C_1 = \frac{1}{r(1+r'^2)^{1/2}},\ C_2 = -\frac{r''}{(1+r'^2)^{3/2}}\ . \tag{4}$$

The mean membrane curvature is :

$$H = \frac{C_1 + C_2}{2} = \frac{-r''r + r'^2 + 1}{2r(1+r'^2)^{3/2}} = \frac{1}{2}\left(-h''(z) + \frac{1}{R} - \frac{h(z)}{R^2}\right)\ , \tag{5}$$

where the curvature was expanded for small deviations from the tube radius, $r(z) = R + h(z)$, $r'(z) = h'(z)$, and $r''(z) = h''(z)$.
The membrane curvature deviator is :

$$D = \frac{C_1 - C_2}{2} = \frac{(1+r'^2) + r''r}{2r(1+r'^2)^{3/2}} = \frac{1}{2}\left(h''(z) + \frac{1}{R} - \frac{h(z)}{R^2}\right)\ , \tag{6}$$

where the curvature was expanded for small deviations from the radius, and where we assumed $C_1$¿$C_1$.

### 0.1 Equilibrium model

The equation of motion of the membrane height deflection (i.e. in the radial direction) along the cylindrical main axis is given by [3, 4]:

$$\varphi\frac{\partial h(z)}{\partial t} = -\frac{\partial F}{\partial h(z)}\ , \tag{7}$$

where $\varphi$ is the friction coefficient describing the drag of the fluid surrounding the membrane, and $F$ is the system free energy (Veksler and Gov, 2007;

Kabaso et al., 2011a). The forces (per unit area) derived from the differentiation of the free energy (Eq.1) are :

$$
\begin{aligned}
-\frac{\partial F}{\partial h(z)} &= -\varphi\kappa\frac{(-2+2{C_{1m}}^2(n^+-1)^2R^2+R^2\sigma)}{4R^3} \\
&+\varphi\kappa\frac{((2+8C_{1m}(n^+-1)R+2{C_{1m}}^2(n^+-1)^2R^2+R^2\sigma)h[z]}{4R^4} \\
&+\varphi\kappa\frac{R^2((2+4C_{1m}(n^+-1)R+2{C_{1m}}^2(n^+-1)^2R^2+R^2\sigma)h''[z]-4R^2h''''[z])}{4R^4} \;. \qquad (8)
\end{aligned}
$$

The fusion pore and the vesicle share the same cation concentration. As a result, the effects of cations on the spontaneous curvature of the fusion pore is uniform throughout the pore.

By a small perturbation to the uniform initial state, we obtain :

$$ r(z) = R + \delta h(z,t) \;, \qquad (9) $$

where $\delta h(z,t)$ is a small deviation from the uniform value.
The linearization of Eq. (7) yields :

$$ \varphi\frac{\partial h(z)}{\partial t} = \int \left(U + \delta L(h) + O(\delta^2)\right) dA \qquad (10) $$

where the function $\delta L(h)$ describes the small undulation in the membrane force, and $O(\delta^2)$ describes higher order terms of the membrane undulation. The force acting on the membrane at equilibrium state is described by $U$. The equilibrium radius $R_{eq}$ is obtained by assuming that the undulation $U$ equals zero. The equilibrium radii of the fusion pore is :

$$ R_{eq} = \frac{1}{\sqrt{{C_{1m}}^2(n^+-1)^2+\sigma/2}} \;. \qquad (11) $$

The infinitesimal area $dA$ of the axisymmetric model is (up to quadratic

order):

$$dA = r(z)\left(1 + \frac{1}{2}h'(z)^2\right) 2\pi \; dz \; . \tag{12}$$

# References

[1] A. Iglič, B. Babnik, U. Gimsa and V. Kralj-Iglič, On the role of membrane anisotropy in the beading transition of undulated tubular membrane structures, J. Phys. A: Math. Gen., 38 (2005) 85278536.

[2] A. Iglič, M. Lokar, B. Babnik, T. Slivnik, P. Veranič, H. Hägerstrand, V. Kralj-Iglič, Possible role of flexible red blood cell membrane nanodomains in the growth and stability of membrane nanotubes, Blood Cells Mol. Dis. 39 (2007b) 14-23.

[3] A. Veksler, N.S. Gov, Phase transitions of the coupled membranecytoskeleton modify cellular shape, Biophys. J. 93 (2007) 3798-3810.

[4] D. Kabaso, R. Shlomovitz, K. Schloen, T. Stradal, N.S. Gov, Theoretical model for cellular shapes driven by protrusive and adhesive forces. PLoS Comp. Biol. 7 (2011a) 1-13.
